# Estimating the effect of corporate integrity culture on tax avoidance using a text-based approach: A research note

**DOI:** 10.1371/journal.pone.0298528

**Published:** 2024-05-14

**Authors:** Pattanaporn Chatjuthamard, Pandej Chintrakarn, Suwongrat Papangkorn, Pornsit Jiraporn

**Affiliations:** 1 Center of Excellence in Management Research, For Corporate Governance and Behavioral Finance, Sasin School of Management, Chulalongkorn University, Bangkok, Thailand; 2 Business Administration Division, Mahidol University International College (MUIC), Salaya, Thailand; 3 Penn State Great Valley School of Graduate Professional Studies, Pennsylvania State University, State College, Pennsylvania, United States of America; Universiti Pertahanan Nasional Malaysia, MALAYSIA

## Abstract

Tax avoidance holds immense importance due to its substantial implications for government revenues and the fair allocation of resources. Consequently, understanding the factors that shape tax avoidance is critically important. Exploiting a cutting-edge measure of corporate integrity derived from state-of-the-art machine learning algorithms and textual analysis, we explore the effect of corporate integrity on tax avoidance. Our text-based measure is based on a textual analysis of earnings conference call transcripts. Our findings show that companies with greater corporate integrity are significantly less involved in tax avoidance. Further analysis corroborates the results, i.e., propensity score matching, entropy balancing, and an instrumental variable analysis. Our findings are especially noteworthy as they demonstrate that corporate culture, although intangible in nature, exerts a substantial influence on corporate behavior.

## I. Introduction

Tax avoidance is a critically important issue due to its significant impact on government revenues and the equitable distribution of resources. Addressing this issue is essential for promoting a fair and sustainable economic system. Not surprisingly, tax avoidance has generated an immense volume of research in accounting, finance, economics, and other areas (see [1] for a literature review). We extend the body of knowledge in this area by investigating how corporate tax avoidance is influenced by one of the most important corporate cultural traits, i.e., corporate integrity.

Corporate culture can be defined as the collective set of beliefs, values, and preferences shared by employees within a corporation [2,3]. Corporate culture is abstract and is difficult to quantify empirically. However, recently, Li et al. [4] employ advanced machine learning algorithms and sophisticated textual analysis of earnings conference calls to identify corporate cultural traits. Using Li et al.’s [4] text-based approach, we explore how a culture of corporate integrity influences corporate tax avoidance.

Firms with a strong culture of integrity should be less involved in corporate tax avoidance. Such firms prioritize transparency, compliance with laws, and ethical decision-making, which extends to their tax practices. Corporate tax avoidance often involves exploiting legal loopholes and engaging in aggressive tax planning to minimize tax liabilities, sometimes at the expense of society’s broader interests. Companies that prioritize integrity are more likely to adhere to the spirit of the tax laws and contribute their fair share of taxes to the society in which they operate [4]. Furthermore, firms with a strong culture of integrity tend to value long-term sustainability and the preservation of their reputation. Engaging in aggressive tax avoidance can be detrimental to a company’s image and could lead to reputational damage if such practices are exposed or criticized by the public, shareholders, or other stakeholders. Therefore, we hypothesize that firms with a stronger culture of integrity exhibit less tax avoidance.

Using a large sample of U.S. firms and a distinctive measure of corporate integrity derived from sophisticated machine learning algorithms and textual analysis, we show that greater corporate integrity results in a significant reduction in tax avoidance, corroborating our hypothesis. To mitigate endogeneity, we perform a variety of robustness checks, i.e., propensity score matching, entropy balancing, and an instrumental variable analysis. All robustness checks validate the results.

Our results extend the existing literature in several important ways. First, we extend the literature on corporate culture. Previous research has predominantly examined corporate culture from a theoretical perspective [2–6]. However, empirical investigations on corporate culture have been limited. Our study addresses this gap and stands as the first empirical research to document that a culture of integrity influences corporate tax avoidance significantly.

In addition to our contribution to the literature on corporate culture, we also enrich the existing research on tax avoidance. Prior studies have primarily focused on accounting or financial factors as determinants of tax avoidance ([1], provide a comprehensive review). Our study aptly augments this body of knowledge by demonstrating that corporate culture, a non-financial and abstract attribute, significantly influences corporate tax avoidance. Finally, we contribute to an emerging area of empirical research that employs textual analysis (see [7], for a literature review). We show that textual analysis can be used to extract abstract quality and create metrics that are empirically relevant and useful.

## II. Prior research on tax avoidance

Our research belongs to a crucial stream of literature that investigates the determinants of tax avoidance. This area holds significant importance and has given rise to an extensive body of research. Early studies primarily concentrated on intrinsic corporate attributes such as company size and operational strategies [1,8–10]. By contrast, contemporary tax avoidance research has integrated corporate governance attributes aimed at mitigating agency conflicts [1,11]. For instance, according to McGuire et al. [12], firms employing dual class share structures tend to exhibit a reduced inclination towards tax avoidance. This could be because insiders hold control over voting rights, potentially alleviating the pressure on management from external shareholders to partake in tax avoidance practices. Richardson et al. [13] find a decline in tax avoidance when the internal audit committee consists of more outside independent directors. External governance mechanisms, such as media exposure, are also relevant to tax avoidance. As discussed in Tian et al. [14]and Kanagaretnam et al. [15], lower levels of tax avoidance are documented when there is strong media exposure of aggressive tax behaviors.

Product market considerations also play a role in tax avoidance. For instance, Kubick et al. [16] highlight that companies leading in the product market tend to employ greater tax avoidance strategies, leveraging their comparative advantage. Conversely, entities possessing valuable brands tend to exhibit lower levels of tax avoidance due to their concerns about maintaining a positive reputation [17]. Several other factors also influence the extent of tax avoidance. Wang et al. [1] offer a contemporary and complete literature review of tax avoidance.

While it is intuitive to assume that corporate integrity is relevant to tax avoidance, there is surprisingly scant research on this issue. One of the reasons is that it is challenging to capture corporate integrity. While corporate integrity is a straightforward and simple concept, it is difficult to operationalize it empirically. We address this gap in the literature by exploiting an innovative measure of a corporate culture of integrity based on advanced algorithms and textual analysis.

## III. Sample selection and data description

Our original sample comes from Li et al. [4]. Then, we combine the data with COMPUSTAT to obtain firm-specific characteristics and financial statement data necessary to estimate tax avoidance measures. The final sample comprises 41,138 firm-year observations from 2001 to 2018.

The measure of corporate integrity used in our study is derived from Li et al. [4], who employ a sophisticated approach to extract phrases pertaining to corporate integrity from earnings conference call transcripts. The frequency of appearance of these phrases serves as an indicator of the level of integrity possessed by the firm. Li et al. [4] execute a variety of validation tests and conclude that their approach is reliable and useful. More information about the construction of the text-based corporate culture score, of which corporate integrity is an important component, is provided in the S1 Appendix.

For tax avoidance, we utilize several alternative measures for robustness, i.e., (1) cash effective tax rate (cash ETR), (2) GAAP effective tax rate (GAAP ETR), (3) book-tax-differences (BTD) and (4) permanent book-tax-differences (Perm-diff). These measures of tax avoidance are widely used in the literature (De Simone et al., 2020). We multiply the cash ETR and the GAAP ETR by minus one in the regression analysis for ease of interpretation. Therefore, a higher value of each of our measures indicates greater tax avoidance.

Additionally, we combine all the four measures discussed above into a single index using principal component analysis (PCA). PCA allows researchers to transform their original variables into a new set of uncorrelated variables, known as principal components. These components are ordered by their ability to explain the variance in the data, with the first component capturing the most variance and subsequent components capturing decreasing amounts. By reducing the dimensionality of our data while retaining the most important information, PCA helps identify the underlying structure and relationships among variables. By focusing on what the four different measures share, we can reduce errors considerably. We referred to the combined measure, which is the first component resulting from PCA, as the tax avoidance index.

We incorporate several firm-specific attributes that may affect tax avoidance. Specifically, we include controls for firm size (natural logarithm of total assets), profitability (EBIT divided by total assets), leverage (total debt divided by total assets), capital investments (capital expenditures/total assets), cash holdings (cash holdings divided by total assets), intangible assets (research and development (R&D) expenses divided by total assets, and advertising expenses divided by total assets), asset tangibility (fixed assets divided by total assets), dividend payouts (total dividends divided by total assets), and discretionary spending (selling, general, and administrative (SG&A) expenses divided by total assets). Additionally, we include industry and year fixed effects to account for variations across industries and over time. It is difficult to include firm fixed effects in the context of our study as corporate culture changes slowly over time, making it challenging to incorporate firm fixed effects. Table 1 shows the summary statistics for the variables.

**Table 1:** Summary statistics.

	Mean	S.D.	25^th^	Median	75^th^
Corporate Integrity	0.575	0.481	0.266	0.469	0.754
Cash ETR	0.176	17.866	0.000	0.123	0.292
GAAP ETR	0.124	11.683	0.000	0.236	0.358
Book-Tax Differences	-0.069	0.396	-0.050	0.012	0.045
Permanent Book-Tax Differences	-0.074	1.194	-0.129	0.115	0.316
Ln(Total Assets)	6.895	2.067	5.460	6.832	8.256
Total Debt/Total Assets	0.247	0.231	0.030	0.212	0.378
EBIT/Total Assets	0.019	0.202	0.006	0.063	0.111
Capital Expenditures/Total Assets	0.051	0.057	0.015	0.032	0.063
R&D/Total Assets	0.054	0.108	0.000	0.001	0.062
Advertising/Total Assets	0.011	0.028	0.000	0.000	0.006
Cash Holdings/Total Assets	0.205	0.226	0.038	0.115	0.295
Dividends/Total Assets	0.014	0.031	0.000	0.000	0.017
SG&A/Total Assets	0.239	0.253	0.052	0.167	0.340
Fixed Assets/Total Assets	0.507	0.424	0.167	0.382	0.781

Essentially, we run the following OLS regression analysis with industry and year fixed effects:

$$\begin{array}{l} Tax\;Avoidance_{it}=\beta_{0}+\beta_{1}{\left(Corporate\;Integrity\;Culture\;Score\right)}_{it}+\\ \beta_{3}{\left(Controls\right)}_{it}+Industry\;Fixed\;Effects+Year\;Fixed\;Effects \end{array}$$

where i indexes firms and t indexes years.

## IV. Results

The results are presented in Table 2. Standard errors are clustered by firm. Model 1 has the cash ETR as the dependent variable. The coefficient associated with corporate integrity is significantly positive at the 10% level, suggesting that corporate integrity results in less tax avoidance. The dependent variable in Model 2 is the GAAP ETR, where the coefficient of corporate integrity is insignificant. Model 3 and Model 4 have book-tax differences and permanent book-tax differences as dependent variables respectively. The coefficients of corporate integrity in Model 3 and Model 4 are significantly negative, implying that greater corporate integrity diminishes tax avoidance. Finally, we use the combined measure of tax avoidance in Model 5 and obtain a significantly negative coefficient for corporate integrity. Overall, our results demonstrate a significant decline in tax avoidance in the presence of higher corporate integrity, consistent with our hypothesis.

**Table 2:** The effect of corporate integrity on tax avoidance.

	(1)	(2)	(3)	(4)	(5)
	Cash ETR	GAAP ETR	BTD	Permanent BTD	Tax Avoidance Index
**Corporate Integrity**	**-0.005** *	**0.003**	**-0.005** ***	**-0.007** *	**-0.022** ***
	**(1.816)**	**(-1.506)**	**(-3.724)**	**(-1.843)**	**(-3.520)**
Ln(Total Assets)	0.012***	0.006***	0.005***	0.009***	0.016***
	(11.941)	(6.925)	(8.613)	(7.714)	(9.033)
Total Debt/Total Assets	-0.074***	-0.054***	-0.088***	-0.342***	-0.493***
	(-9.296)	(-8.580)	(-20.512)	(-29.995)	(-27.073)
EBIT/Total Assets	0.190***	0.256***	0.824***	3.029***	4.623***
	(17.732)	(32.752)	(98.381)	(155.143)	(130.186)
Capital Expenditures/Total Assets	-0.207***	0.008	-0.004	0.187***	0.227***
	(-6.743)	(0.310)	(-0.234)	(4.946)	(3.730)
R&D/Total Assets	-0.212***	-0.025	-0.128***	-0.041	-0.269***
	(-7.760)	(-1.464)	(-8.597)	(-1.095)	(-3.733)
Advertising/Total Assets	-0.023	0.081	-0.086**	0.043	-0.138
	(-0.370)	(1.574)	(-2.158)	(0.516)	(-1.212)
Cash Holdings/Total Assets	-0.096***	-0.129***	0.015***	0.123***	0.127***
	(-10.042)	(-17.338)	(3.086)	(10.248)	(6.422)
Dividends/Total Assets	0.049	-0.313***	-0.246***	0.425***	-0.056
	(1.103)	(-8.004)	(-7.042)	(5.663)	(-0.486)
SG&A/Total Assets	0.067***	0.010*	-0.048***	-0.090***	-0.210***
	(7.494)	(1.647)	(-9.724)	(-7.344)	(-10.561)
Fixed Assets/Total Assets	-0.002	-0.001	0.010***	0.000	0.021**
	(-0.437)	(-0.181)	(3.976)	(0.028)	(2.140)
Constant	0.150***	0.249***	-0.054***	-0.068***	-0.140***
	(16.045)	(33.051)	(-11.326)	(-5.936)	(-8.003)
Industry Fixed Effects	Yes	Yes	Yes	Yes	Yes
Year Fixed Effects	Yes	Yes	Yes	Yes	Yes
Observations	33,030	41,138	37,422	37,257	32,784
R-squared	0.141	0.283	0.781	0.845	0.767

Robust t-statistics in parentheses.

*** p<0.01,

** p<0.05,

* p<0.1.

To minimize endogeneity, we run several robustness checks and show the results in Table 3. First, we perform propensity score matching (PSM). We classify firms in the top quartile of corporate integrity as our treatment group. For each observation in the treatment group, we select an observation in the rest of the sample that is most similar using ten firm-specific attributes (the ten control variables in the regression analysis). Hence, our treatment and control groups are indistinguishable in every observable dimension, except for corporate integrity. Model 1 in Table 3 contains the PSM result, showing a significantly negative coefficient for corporate integrity.

**Table 3:** Robustness checks.

	(1)	(2)	(3)	(4)
	PSM	Entropy Balancing	IV (1)	IV (2)
	Tax Avoidance Index	Tax Avoidance Index	Tax Avoidance Index	Tax Avoidance Index
**Corporate Integrity**	**-0.019** **	**-0.016** **	**-0.106** ***	**-0.047** **
	**(-2.375)**	**(-2.229)**	**(-3.094)**	**(-2.255)**
Ln(Total Assets)	0.016***	0.018***	0.010***	0.011***
	(6.056)	(8.109)	(2.827)	(3.055)
Total Debt/Total Assets	-0.516***	-0.501***	-0.731***	-0.731***
	(-16.773)	(-19.801)	(-28.089)	(-28.083)
EBIT/Total Assets	4.607***	4.661***	5.988***	6.006***
	(79.711)	(103.566)	(143.792)	(147.456)
Capital Expenditures/Total Assets	0.108	0.121	-0.110	-0.092
	(0.940)	(1.287)	(-0.854)	(-0.719)
R&D/Total Assets	-0.346***	-0.210**	-0.900***	-0.887***
	(-3.188)	(-2.530)	(-9.992)	(-9.871)
Advertising/Total Assets	-0.307	-0.326**	-0.775***	-0.744***
	(-1.557)	(-1.986)	(-3.887)	(-3.747)
Cash Holdings/Total Assets	0.128***	0.122***	0.443***	0.440***
	(3.793)	(4.687)	(13.014)	(12.940)
Dividends/Total Assets	0.070	-0.069	-0.915***	-0.942***
	(0.353)	(-0.463)	(-4.764)	(-4.915)
SG&A/Total Assets	-0.162***	-0.169***	-0.427***	-0.429***
	(-5.545)	(-6.872)	(-13.985)	(-14.051)
Fixed Assets/Total Assets	0.035**	0.031**	0.047**	0.050***
	(2.158)	(2.120)	(2.523)	(2.695)
Constant	-0.149***	-0.168***	-0.150	-0.182
	(-5.400)	(-7.399)	(-0.903)	(-1.101)
Industry Fixed Effects	Yes	Yes	Yes	Yes
Year Fixed Effects	Yes	Yes	Yes	Yes
Observations	13,969	32,784	33,452	33,452
R-squared	0.809	0.792	0.565	0.565

Robust t-statistics in parentheses.

*** p<0.01,

** p<0.05,

* p<0.1.

In Model 2, we execute entropy balancing, where we adjust the weight of each observation such that the means and the variances of the treatment and control groups are comparable. Again, the coefficient of corporate integrity is significantly negative. Furthermore, we implement an instrumental-variable analysis (IV). Our first instrument is the value of corporate integrity in the earliest year for each firm. Since corporate integrity in the earliest year could not have resulted from corporate integrity in any of the subsequent years, reverse causality is mitigated. We conduct an instrumental-variable analysis in two stages. Initially, we regress the corporate integrity score on our instrumental variable and all control variables, subsequently saving the predicted value of the corporate integrity score. In the second stage, we regress tax avoidance on this predicted value and the control variables. This approach aligns with the standard procedure for instrumental variable analyses. Model 3 is the second-stage regression result, where the coefficient of corporate integrity instrumented from the first stage is significantly negative. The Shea partial R^2^ for our first-stage regression stands at 30.89%, and with an F-statistic of 14,907.08, it is clear that our instrument is robust and statistically significant.

Finally, we employ another instrumental variable. Due to investors’ clientele, local competition, and social interactions, companies located nearby tend to share similar characteristics, including corporate culture [18]. Our second instrumental variable is the average value of corporate integrity of all firms located in the same city. The value at the city level should influence corporate integrity at the firm level but is not directly correlated with firm-specific tax avoidance because there are many firms in the same city. Again, we carry out a two-stage instrumental-variable analysis. First, we regress the corporate integrity score against our instrumental variable and control variables, then save the predicted score. In the second stage, we regress tax avoidance against this predicted score and control variables. Model 4 shows the second-stage regression, where the coefficient of corporate integrity is significantly negative. The first-stage regression has a Shea partial R^2^ of 30.89% and a highly statistically significant F-statistic of 14,907.08, confirming the robustness of our instrument. Overall, there is robust evidence that companies with stronger corporate integrity are significantly less aggressive in avoiding taxes, corroborating our hypothesis.

## V. Conclusions

We hypothesize that companies with a stronger culture of integrity should be less involved in tax avoidance. Based on a unique measure of corporate integrity generated by cutting-edge machine learning algorithms and textual analysis, our findings show that greater corporate integrity brings about a significant reduction in corporate tax avoidance, corroborating our hypothesis. Additional robustness checks validate the results, i.e., propensity score matching, entropy balancing, and an instrumental variable analysis. Our findings are crucially important as they demonstrate the tangible influence of corporate culture on corporate behavior, despite its abstract nature.

Our research findings carry significant practical implications for diverse stakeholders. First, shareholders and managers gain insights into the significance of corporate culture, despite its abstract nature, as it correlates with corporate behavior. Consequently, promoting corporate integrity becomes imperative. Secondly, regulators and policymakers can utilize our findings to consider the impact of corporate culture while formulating tax-related regulations. This understanding can lead to more effective and targeted policy measures. Thirdly, investors benefit from our research by recognizing the relevance of corporate culture in influencing corporate behavior. This awareness enables them to make more informed and accurate assessments of companies. Lastly, tax authorities can leverage our findings to make well-informed decisions on reducing tax avoidance. In summary, our study provides actionable insights for stakeholders to foster corporate integrity, create effective tax policies, make informed investment decisions, and address tax avoidance challenges.

Finally, a couple of limitations of our research can be noted. First, corporate integrity has a broad meaning. We use a text-based measure of corporate integrity culture as a proxy for corporate integrity. While our measure is useful in capturing the extent of corporate integrity, some aspects of corporate integrity may be left out. One way to address this limitation is for future research to utilize other proxies for corporate integrity that may reflect other aspects of corporate integrity. Second, while we employ several proxies for tax avoidance for robustness, there are a few other proxies that are not included. Future researchers may extend our study by including additional measures of tax avoidance. For instance, recently, a new measure of tax avoidance called “uncertain tax positions” has been examined in the literature. This new measure is enabled by the Accounting Standard Codification (ASC) 740. It would be interesting for future research to explore the impact of corporate integrity using this new proxy for tax avoidance.

## Supporting information

S1 Appendix (DOCX)

## References

[1] Wang F, Xu S, Sun J, Cullinan CP. Corporate Tax Avoidance: A Literature Review and Research Agenda. J Econ Surv 2020;34:793–811. doi: 10.1111/joes.12347

[2] Crémer J. Corporate Culture and Shared Knowledge. Industrial and Corporate Change 1993;2:351–86. doi: 10.1093/icc/2.3.351

[3] Van den Steen E. On the origin of shared beliefs (and corporate culture). Rand J Econ 2010;41:617–48. doi: 10.1111/j.1756-2171.2010.00114.x

[4] Li K, Mai F, Shen R, Yan X. Measuring Corporate Culture Using Machine Learning. Rev Financ Stud 2021;34:3265–315. doi: 10.1093/rfs/hhaa079

[5] Weber Y, Shenkar O, Raveh A. National and Corporate Cultural Fit in Mergers/Acquisitions: An Exploratory Study. Manage Sci 1996;42:1215–27. doi: 10.1287/mnsc.42.8.1215

[6] Graham JR, Grennan JA, Harvey CR, Rajgopal S. Corporate culture: The interview evidence. Journal of Applied Corporate Finance 2022;34:22–41. doi: 10.1111/jacf.12528

[7] Loughran T, Mcdonald B. Textual Analysis in Accounting and Finance: A Survey. Journal of Accounting Research 2016;54:1187–230. doi: 10.1111/1475-679X.12123

[8] Rego SO. Tax-Avoidance Activities of U.S. Multinational Corporations. Contemporary Accounting Research 2003;20:805–33. doi: 10.1506/VANN-B7UB-GMFA-9E6W

[9] Wilson RJ. An Examination of Corporate Tax Shelter Participants. The Accounting Review 2009;84:969–99. doi: 10.2308/accr.2009.84.3.969

[10] Lisowsky P. Seeking Shelter: Empirically Modeling Tax Shelters Using Financial Statement Information. The Accounting Review 2010;85:1693–720. doi: 10.2308/accr.2010.85.5.1693

[11] Hanlon M, Heitzman S. A review of tax research. Journal of Accounting and Economics 2010;50:127–78. doi: 10.1016/j.jacceco.2010.09.002

[12] McGuire ST, Wang D, Wilson RJ. Dual Class Ownership and Tax Avoidance. The Accounting Review 2014;89:1487–516. doi: 10.2308/accr-50718

[13] Richardson G, Taylor G, Lanis R. The impact of board of director oversight characteristics on corporate tax aggressiveness: An empirical analysis. Journal of Accounting and Public Policy 2013;32:68–88. doi: 10.1016/j.jaccpubpol.2013.02.004

[14] Tian G, Si Y, Han J, Bian Y. Media coverage and tax aggressiveness: A study from the perspective of corporate governance. Journal of Management Science and Engineering 2016;29:104–21.

[15] Kanagaretnam K, Lee J, Lim CY, Lobo G. Societal trust and corporate tax avoidance. Review of Accounting Studies 2018;23:1588–628. doi: 10.1007/s11142-018-9466-y

[16] Kubick TR, Lynch DP, Mayberry MA, Omer TC. Product Market Power and Tax Avoidance: Market Leaders, Mimicking Strategies, and Stock Returns. The Accounting Review 2015;90:675–702. doi: 10.2308/accr-50883

[17] Austin CR, Wilson RJ. An Examination of Reputational Costs and Tax Avoidance: Evidence from Firms with Valuable Consumer Brands. Journal of the American Taxation Association 2017;39:67–93. doi: 10.2308/atax-51634

[18] Jiraporn P, Jiraporn N, Boeprasert A, Chang K. Does Corporate Social Responsibility (CSR) Improve Credit Ratings? Evidence from Geographic Identification. Financ Manage 2014;43:505–31. doi: 10.1111/fima.12044

