# Supplementary material for: Estimating the effect of corporate integrity culture on tax avoidance using a text-based approach: A research note
Source: PLoS One. 2024 May 14;19(5):e0298528. doi: 10.1371/journal.pone.0298528 (PMC11093333; doi:10.1371/journal.pone.0298528)
Supplement: S1 Appendix — (DOCX) [file pone.0298528.s001.docx]

**Appendix**

**Description of the construction of the text-based corporate culture score**

Leveraging an extensive array of 209,480 earnings calls from the Thomson Reuters’ Street Events database, spanning the years 2001 to 2018, Li et al. (2021) meticulously train a word embedding model, utilizing this advanced linguistic tool to decode and quantify the corporate cultural values embedded within 7,501 distinct firms. This endeavor yields a comprehensive set of 62,664 firm-year observations, providing a rich tapestry of corporate cultural landscapes.

To ensure the robustness of their corporate culture metrics, Li et al. (2021) rigorously benchmark them against well-respected standards in pivotal areas such as corporate innovation, integrity, product excellence, respect, and teamwork. Their analysis reveals a striking and significant positive correlation between their derived cultural values and these esteemed markers, underscoring the validity of their approach.

Additionally, the research involves a comparative evaluation of their primary metric, which focuses primarily on the Question and Answer (Q&A) segments of earnings calls, with several alternative methodologies. These alternative approaches include: (a) a comprehensive analysis of entire earnings calls, encompassing both the management presentation and Q&A sections; (b) a basic enumeration of specific seed words as identified in Guiso, Sapienza, and Zingales’ (2015) research, particularly those occurring within the Q&A discourse; and (c) the application of the word embedding model to the Management’s Discussion and Analysis (MD&A) segments found in annual reports, commonly referred to as 10-Ks.

Upon conducting these comparative evaluations, their findings compellingly demonstrate that the application of the word embedding model specifically to the Q&A sections of earnings calls significantly enhances the accuracy and depth of measuring corporate culture. This methodological refinement represents a noteworthy advancement over the previously mentioned alternative approaches, offering a more nuanced and insightful assessment of the complex tapestry of corporate culture.
